# Teaching Life Writing in a Blended Learning Environment

**DOI:** 10.1080/08989575.2018.1390042

**Published:** 2017-11-09

**Authors:** Sarah Herbe

**Affiliations:** ^a^ University of Salzburg

## Introduction

In order to introduce the topic, I once started a course on paratexts by distributing two editions of a text to small groups of students, asking them to discuss the following questions: what do you learn about the book simply by looking at its cover, the back cover, and the first pages, as well as its material makeup? Based on these features, what do you think that the book is about? And how do the two editions differ from each other? One of the pairs of books I distributed was the two editions of Sidonie Smith and Julia Watson's *Reading Autobiography: A Guide for Interpreting Life Narratives*, the different external presentation of which had always surprised me. While the responses of two of the three members of the group dealing with *Reading Autobiography* complied with the reactions I had anticipated—they commented on the different expectations raised by the black cover of the newer edition, dominated by the letter “A,” suggesting an authoritative academic text, as opposed to the two shades of mint-green stripes that dominate the cover of the first edition, reminiscent of bedroom wallpaper, which rather evokes the private aspect of autobiographical writing—the reaction of the third student to the question of what these books could be about astonished me: “Why would you write a whole book about autobiographies? What is there to say about autobiographies? Isn't autobiography the most straightforward genre there is?” While this reply was to a certain extent sobering for a teacher of a class of advanced students of anglophone literatures and cultures, it served to remind me of the attitude that students, and readers in general, might bring to life-writing texts, and I tried to stay aware of this attitude when planning courses on various aspects of life writing. In the following, I shall focus on the first course on life writing I ever taught: a seminar on women's life writing held in the winter term of 2010–11 in the Department of English and American Studies at the University of Salzburg, Austria, as part of a module called “Aspects of Cultural Studies.” This course introduced undergraduate students to a number of critical approaches toward life writing that they would then apply to a deliberately diverse corpus of life-writing texts by women, ranging from *The Book of Margery Kempe* to Jeanette Winterson's online column. One of the aims of this type of course in the curriculum is to equip students with an inventory of research skills and analytical strategies in order to prepare them for writing their final theses in the field of literary or cultural studies.

The seminar comprised fourteen sessions from October to January (ninety minutes of contact time per week) and consisted of three phases. In the first phase, previous knowledge was activated and students were introduced to basic theoretical approaches toward life writing. In the second phase, students applied their knowledge and analytical skills to one of the set texts and presented their results to the class. Finally, their results were further elaborated in a final essay, to be submitted at the end of the semester. Grading was based on written and oral contributions over the course of the semester, as well as the final essay.

The seminar was open to a variety of students: it was an elective course for Bachelor's students of English studies in their second, third, or fourth semester, as well as for teacher-training students in their third and fourth years of study, which resulted in a very heterogeneous group of students. While some Bachelor's students were at the very beginning of their studies, with hardly any previous knowledge about literary or cultural theory, for some of the teacher-training students this course was among the last they attended before completing their studies, during which they had already been exposed to a number of classes on literary and cultural studies.

All courses at the University of Salzburg are automatically supported by the virtual learning environment Blackboard, but the teacher ultimately decides which features of this online platform will be employed for each course. Since, for many students, the seminar on life writing was basically their first contact with the topic, and for some even the first exposure to literary and cultural theory, I decided to employ discussion forums and blog entries in order to enable profound discussions and facilitate preparation for tasks in class, as well as promote the students’ communicative skills and enable constructive peer-reviewing. Such a blended learning scenario, in which “a face-to-face (F2F) classroom component [is combined] with an appropriate use of technology” (Barrett and Sharma 7), not only fosters students’ thorough engagement with required reading and presents a possibility for mapping their research efforts, but also caters to the needs of different student types. Asynchronous tools such as online discussion boards, for example, allow students to formulate questions about complex texts or issues at their own speed and “may encourage attention to accuracy as well as ‘deeper’, more considered thinking than in synchronous exchanges” (107). Further, blended learning works well in a heterogeneous classroom such as the one I was confronted with, since it allows the teacher to gauge the different levels of previous knowledge that students bring to class, and therefore facilitates targeted preparation.

## Course Outline

In order to make explicit the genre expectations we often bring to life writing, I started the first session with a brief account of Margaret B. Jones's *Love and Consequences: A Memoir of Hope and Survival*, which had been exposed as fake not long after its publication (Rich). Informing the students about the strong reactions to this revelation—the copies of the book were recalled, people got their money back, and Jones's book tour was canceled (Rich)—I asked them to think about why the fact that the memoir was faked was perceived as such a scandal, thereby initiating a discussion about truth in life writing as well as the supposed identity of author, narrator, and protagonist. In the framework of this discussion, I introduced some of the basic questions and tenets of life-writing criticism, drawing on Philippe Lejeune's “The Autobiographical Pact,” Paul John Eakin's *Touching the World*, and Smith and Watson's *Reading Autobiography*. In small groups, the students would then discuss whether they had read any life-writing texts so far and whether women were the authors of any of those texts. Sharing the results of this discussion with the group then served to introduce the individual students to the group and as a basis for reading a number of paragraphs from “Sixty Genres of Life Narrative,” by Smith and Watson, which introduced students to the particular genres of life writing they would encounter over the course of the semester (spiritual life narrative, diary, digital life stories, and memoirs).

After the first session, students were asked to read selected chapters from Linda Anderson's *Autobiography* and Susan Stanford Friedman's “Women's Autobiographical Selves: Theory and Practice” with the help of guiding questions that particularly addressed the notion of selfhood and gender consciousness in life writing, then to write a brief blog entry on Blackboard on any life-writing text by a woman. This could either be a text they were already familiar with or any text they encountered in the course of Internet research or, alternatively, with the help of the bibliography on the history and theory of life writing distributed in the first session. They were assigned to read the first pages of the chosen texts and describe at what point in the writer's life the narrative begins (family history, childhood, etc.), how it begins, what we learn about its structure, and whether the reader is told why it was written. This blog entry was due before the next class meeting, and students were encouraged to read their colleagues’ posts in order to get an idea of the variety of life writing by women, as well as their possible reasons for writing, which would form the basis for discussing models of emplotment as well as the issue of coaxers (see Smith and Watson 2010 64–69) in the second session.

Knowledge of several critical approaches toward women's life writing was acquired in the form of group presentations in the third session of the semester. In groups of four or five (seventeen students attended the class), students were asked to prepare an oral presentation of ten minutes on a particular article,[Fn en0001] including the main argument of the critic, the three most important observations of the article, and a brief discussion about which ideas they found particularly convincing and which were rather puzzling. Since the preparation time for these short presentations was limited, and students often have difficulties finding time for meetings to prepare their presentations together, I encouraged them to make use of online tools for working on a document together (such as Google Docs), as well as group discussions with the help of social media. The presenters prepared handouts for all participants so that, at the end of the session, everyone was equipped with an inventory of ideas about approaching women's life writing. This inventory was further expanded by a thorough engagement with the chapters “Autobiographical Acts” and “Autobiographical Subjects” in Smith and Watson.

As other scholars have observed (e.g. Bloom; Drake), Smith and Watson's tool kit as well as their chapters on “Autobiographical Acts” and “Autobiographical Subjects” are indispensable sources for approaching life-writing texts in a teaching environment. Because of the heterogeneity of the participants, I have found it useful to prepare these chapters in online discussion forums. Students were asked to read the chapters at home and to take notes as to what they found particularly useful, intriguing, or perplexing. Before our next meeting in class, they had to contribute at least two posts to the online discussion forum: one pointing out what they found most interesting and one formulating a question about the text. In addition to their own two posts, students had to react to at least one of their colleagues’ contributions. This online discussion made it necessary for students to engage thoroughly with the chapters and forced them to formulate their own opinions toward critical approaches, and it allowed me to know beforehand which ideas or concepts posed particular problems and needed in-depth discussion in class. The obligation to react to at least one of the posts also meant that some questions were answered before class. The assignment to think about what was difficult to grasp in the text—based as it was on the assumption that it is normal to encounter difficulties when we read a (theoretical) text—made it easier for students to overcome their inhibitions to ask questions about anything that was unclear to them. The online discussion environment further meant that students who were too shy to ask questions or contribute to discussions in class could voice their ideas nevertheless (usually they would then become more active in the classroom, too). One of the concepts that posed particular difficulties was “prosthetic memory.” Knowing about this difficulty beforehand allowed me to bring additional sources to class, and, in a productive discussion, one of the students suggested that thinking about prosthetic memory as memory created by “mass-media information” was helpful for understanding the idea. Questions about the analytical category of “voice” allowed us to reflect on how women try to make themselves and their concerns heard by writing their lives. In general, students responded very well to these pre-class online activities—they were usually eager to read their colleagues’ posts and to react to them, and they often contributed more than the required posts—and perceived them as a good preparation for discussions in class, where any open questions would then be answered together, and a list of useful concepts for approaching life-writing texts would be compiled.

In a next step, students would apply their newly acquired knowledge in the form of short comments on the blog entries they had posted at the beginning of the semester: they were asked to write comments both on their own blog posts and on at least one of the others, thinking about which concepts would be suitable for approaching the respective texts. This exercise made them think about the texts they had originally chosen from a new, critical perspective, honed their analytical skills, and prepared them for finding an approach to the course text they had chosen for their oral presentations and final essays. In class, students were asked to get together in groups with those who had chosen the same text in order to discuss possible approaches. In this group work, students further practiced the application of concepts gleaned from Smith and Watson's chapters, and made sure that the topics they chose for the oral presentations would not overlap too much. In order to foster engagement with the course texts, the students were encouraged to bring in perspectives informed by their previous knowledge, second subjects (all teacher-training students in Austria need to enroll for at least two subjects), or, if applicable, professional backgrounds. A student with medical training, for example, focused on medical approaches to *The Book of Margery Kempe*.

The oral presentations were modeled on sessions of academic conferences: twenty minutes of presentation followed by ten minutes of discussion, although students could opt for a discussion at the beginning or in the middle of their presentations as well. This flexibility allowed students to start their presentation with questions to their audience or maintain their colleagues’ attention by discussing a particular point with them before they presented their own results or conclusions. Since there were sometimes three individual presentations in one session, variations of the set pattern presented a welcome diversity and made it easier for students to stay focused on their colleagues’ contributions. For every presentation, one student assumed the role of “chair.” The chair would be particularly well prepared, monitor the time, introduce the topic, and lead the discussion. In order to make sure that all students would actively engage with the presentation topic (and read the course texts thoroughly), the presenters sent out questions on their topics the week before the presentation, and their colleagues were asked to submit their answers two days before the presentation via the “safe assignment tool” in the online environment. Submissions contributed via this tool are visible only to the instructor of the course. This tool was chosen in order to guarantee that all students thought about the questions on their own and would not allow themselves to be influenced by those who submitted their answers first. I would then forward the collected replies to the presenter and the chair, who could react to them in the discussion or even during the presentation. I encouraged the presenters to ask questions they did not necessarily know the answers to in order to give them the possibility to get additional input on their research questions, the approach they had chosen, and the overall topic of their final essay. One student wanted to know whether Smith and Watson's statement that “The conscious diffraction of times of telling and the fragmentation of chronological sequence are narrative means of emphasizing that a subject is not unified or coherent” (2nd Ed. 94) could be applied fruitfully in her discussion of patterns of emplotment in Virginia Woolf's “A Sketch of the Past.” Another student was wondering why at the beginning of the second book of *The Book of Margery Kempe*, Kempe's adult son is referred to so prominently, which initiated a discussion about the importance of relationality in women's life writing, as well as the different (ideological) roles Kempe is presented as assuming in the text. A student who had chosen to talk about how social conventions of the Regency era were manifest in the *Journals and Letters of Frances Burney* wanted to know from her colleagues, “Which, if any, of the social customs and rules of Burney's time still remain applicable today? Which seem completely foreign and strange to you?”

In an effort to make the topic of our seminar further relevant to the students’ (everyday) lives, the presentations on the course texts were supplemented by discussions on current questions of life writing that I or the students stumbled across in the news or other realms of our daily lives. When we dealt with Burney's *Journals and Letters*, we listened to the song “We Used to Wait” by Arcade Fire (which includes the lines, “It seems strange | How we used to wait for letters to arrive”), which initiated a reflection on the changing practices of letter writing in the twenty-first century in the light of online communication. For such discussions, it was most helpful that our group did not consist only of students in their early twenties who had hardly ever composed or received a personal, handwritten letter, but that there were also students in their early thirties and fifties, which resulted in different approaches toward, and memories of, letter writing. Our discussion about online communication led to the sudden insight that all of those students who were on Facebook were, consciously or unconsciously, constantly writing their lives as part of their daily routines. This insight was confirmed by a message Facebook sent to its users in early 2011—namely, that “In the next few days you'll be upgraded to the new profile, which offers more ways to show and tell your story.” This message further made us reflect on how social-media tools such as Facebook determine the way we tell stories about our lives and how we present ourselves to the outside world. This debate prepared the ground for the discussion of our final course text, Winterson's online column, which the British author published in blog form as part of her personal website from 2000 to 2014.[Fn en0002] Winterson's column occasioned a lively discussion about the differences between digital and non-digital life writing, with one student asking the group, “How would Jeanette Winterson's writing have been different if she had published it as a hardcover book instead of a blog? Think about her style, the content of her entries, and the editing phase.”

## Concluding Remarks

The frequent opportunities students were given during the semester, both in class and in the online learning environment, to discuss the newly encountered critical concepts, as well as the regular teacher and peer feedback they received over the course of the semester, helped them find fruitful approaches to the texts they had chosen to work on and to write well-researched and original final essays. Since the use of e-learning tools in a blended environment reflects the ubiquity of online expression in the twenty-first century, their use further allowed me to draw attention to the fact that “Nowhere is the power and diversity of the autobiographical more visible than online, where it is the raison d’être for many of the activities and practices associated with Web 2.0” (Poletti and Rak 3). Debating digital life writing in connection with one's own private and professional use of online tools further led to a discussion of the kind of material that (future) life writers will have at their disposal in the light of changing habits of self-documentation, and it made us ponder the advantages and disadvantages of the fact that “we rely more than ever on computer memory to enhance our human capabilities to remember and recall information, both institutional and personal” (Arthur 189).[Fn en0003] Such discussions are not only useful for exploring how life writing has changed over the course of centuries, and how the different tools and materials that life writers have at their disposal affect their self-presentation, but they also allow us to highlight the challenges online self-presentation poses for life-writing scholarship in the twenty-first century.

## Notes

1. The articles chosen for these presentations were by Brée, Juhasz, Peterson, and Watson. This type of group work was modeled on an exercise my colleague Markus Oppolzer introduced in one of his courses.

2. For a detailed discussion of Winterson's column, see my forthcoming essay, “Online Self-Presentation and Promotion in Jeanette Winterson's Column (2000–2014).”

3. I became painfully aware of the potential transitoriness of digital memory when I discovered that Blackboard courses are irrecoverably deleted after two and a half years, meaning that for this essay I had no access to the original blog entries or the discussion forum but had to rely on my conventional notes, files, and occasional printouts, as well as the emails one of my students (Julia Hartinger) had fortunately saved.
